# Intraoperative flap perfusion of radial free forearm flaps is associated with postoperative flap revision in microvascular head and neck reconstruction: a retrospective cohort study

**DOI:** 10.3389/froh.2026.1910824

**Published:** 2026-09-11

**Authors:** Mark Ooms, Philipp Winnand, Marius Heitzer, Nils Vohl, Marie Sophie Katz, Johannes Bickenbach, Frank Hölzle, Ali Modabber

**Affiliations:** 1 Department of Oral and Maxillofacial Surgery, University Hospital RWTH Aachen, Aachen, Germany; 2 Department of Intensive Care Medicine, University Hospital RWTH Aachen, Aachen, Germany

**Keywords:** blood flow, flap perfusion, flap revision, hemoglobin concentration, hemoglobin oxygen saturation, microvascular head and neck reconstruction, radial free forearm flaps

## Abstract

**Background:**

Postoperative vascular flap compromise continues to occur in microvascular head and neck reconstruction, requiring time-consuming postoperative flap monitoring. This study assessed the association between intraoperative flap perfusion and postoperative flap revision.

**Methods:**

Flap perfusion measurement data collected intraoperatively with the Oxygen-2-see analysis system from 296 patients reconstructed with a radial free forearm flap in the head and neck region between 2011 and 2022 were retrospectively analyzed. Intraoperative flap blood flow, hemoglobin concentration, and hemoglobin oxygen saturation at 8 and 2 mm tissue depth were compared between patients without and with postoperative flap revision and cut-off values predicting postoperative flap revision were determined.

**Results:**

Intraoperative flap blood flow and hemoglobin oxygen saturation were lower and hemoglobin concentration was higher in patients with postoperative flap revision compared to patients without postoperative flap revision at 8 mm tissue depth [82.0 arbitrary units (AU) vs. 132.0 AU, *p*<0.001; 67.0% vs. 79.0%, *p* = 0.006; and 53.0 AU vs. 45.0 AU, *p* = 0.025]. The related cut-off values predicting flap revision for flap blood flow, hemoglobin concentration, and hemoglobin oxygen saturation were <82.5 AU, >51.5 AU, and <78.5% (sensitivity 56%, 56%, and 78%; specificity 90%, 69%, and 52%).

**Conclusions:**

Intraoperative flap perfusion was associated with postoperative flap revision, with cut-off values predicting postoperative flap revision determined as minimum blood flow and hemoglobin oxygen saturation and maximum hemoglobin concentration values. These findings should be confirmed in further studies to support the implementation of prevention strategies such as intraoperative anastomosis check and closer postoperative flap monitoring for flaps at risk.

## 1 Introduction

Microvascular reconstruction of the head and neck region offers functionally and aesthetically beneficial outcomes and high success rates (1, 2). However, free flap failure occurs, requiring additional surgical procedures to avoid permanent functional and aesthetic impairment (1, 3).

Postoperative flap monitoring to timely detect vascular flap compromise is therefore essential in microvascular reconstruction, as the time interval between the occurrence and correction of vascular flap compromise is related to flap failure (1, 4–6). Several methods are used for postoperative flap monitoring, including the Oxygen-2-see (O2C) analysis system (LEA Medizintechnik, Germany), which is able to timely detect vascular flap compromise by measuring flap perfusion in relation to predefined threshold values (4, 7–9). However, postoperative flap monitoring only covers the period after the initial surgical procedure and cannot prevent vascular flap compromise with the need for an additional surgical procedure, is time-consuming, and its frequency is still under debate (5, 10–12).

Therefore, predicting postoperative flap revision intraoperatively would be beneficial for the implementation of primary and secondary prevention strategies, i.e., immediate intraoperative anastomosis revision and more frequent postoperative flap monitoring, respectively (13–16). Since flap perfusion is a prerequisite for flap viability and decreased flap perfusion has been linked to flap failure, intraoperative measurement of flap perfusion may be suitable for this purpose (4, 7, 8, 14). Interestingly, a previous study evaluated intraoperative flap perfusion as a risk factor for postoperative flap revision and found that not intraoperative absolute values but only postoperative decreasing trends in perfusion parameter values were indicative of postoperative flap revision (17). However, intraoperative flap measurement was not performed after flap reperfusion, different flap types were combined, and potential confounding variables such as for example blood pressure not considered, making definitive conclusions difficult (17, 18).

The aim of this retrospective study was to quantify the association between intraoperative flap perfusion and postoperative flap revision and evaluate the predictability of postoperative flap revision based on intraoperative flap perfusion measurement.

## 2 Materials and methods

### 2.1 Study design and setting

This retrospective cohort study was conducted at the Department of Oral and Maxillofacial Surgery, University Hospital RWTH Aachen, Germany. The study protocol was approved by the local ethics committee of the Medical Faculty RWTH Aachen University (EK 22–359). Informed consent was waived due to the retrospective design of the study and the use of anonymized data, which were collected as part of routine clinical practice between 2011 and 2022. All procedures were performed in accordance with the institutional ethical standards and with the 1964 Helsinki Declaration and its later amendment.

### 2.2 Patient selection

The study population consisted of 296 patients who underwent microvascular reconstruction of the head and neck region with a radial free forearm flap (RFFF) for malignant or nonmalignant diseases in our Department of Oral and Maxillofacial Surgery between 2011 and 2022. These patients were selected from a total of 333 patients reconstructed with an RFFF, taking into account both the inclusion criteria - complete data sets on clinical data and flap perfusion measurement data - and the exclusion criteria - incomplete data sets.

### 2.3 Data collection

Data were extracted from clinical records. Comorbidities were recorded according to discipline-specific guidelines, and smoking status was recorded according to commonly used definitions as actual or previous daily smoking for a period of at least a six months at the time of the pre-anesthesia interview (19). In terms of pretreatment, the status of prior neck dissection or irradiation was defined as positive in patients who had undergone neck dissection with an anatomic dissection of the recipient vessel or neck irradiation including the area of the recipient vessel at any time before the actual surgery. The duration of surgery was determined as the time interval between the first incision and the last suture, and the duration of flap ischemia was determined as the time interval between the interruption of flap perfusion at the donor site and the onset of flap perfusion after anastomosis at the recipient site. Flap revision was categorized as positive if patients had undergone surgical revision of the anastomosis with return to the operating room, and flap success was categorized as negative if patients had the flap removed due to flap necrosis.

### 2.4 Treatment protocol

All patients underwent microvascular head and neck reconstruction with an RFFF under general anesthesia, with the anastomoses performed manually with sutures, and were monitored postoperatively in the intensive care unit with invasive mechanical ventilation and analgosedation at least until the next morning. Blood pressure was monitored with an invasive arterial catheter and regulated by central venous vasopressor administration (norepinephrine) as needed (with a target systolic pressure above 125 mmHg). The postoperative anticoagulation protocol included subcutaneous injections of heparin (5000 IU) three times a day for 7 days. The microvascular flaps were initially measured intraoperatively and regularly monitored postoperatively with the O2C analysis system. The decision to revise a flap was (independently from this study) based on flap perfusion thresholds that had been identified in previous studies using the O2C analysis system as indicators of vascular flap compromise in RFFFs [blood flow <20 arbitrary units (AU) at 8 mm tissue depth and <10 AU at 2 mm tissue depth, hemoglobin concentration increase >30% at 8 and 2 mm tissue depth, and hemoglobin oxygen saturation <15% at 8 and 2 mm tissue depth] (7, 8).

### 2.5 Flap perfusion measurement data

**Figure 1:**
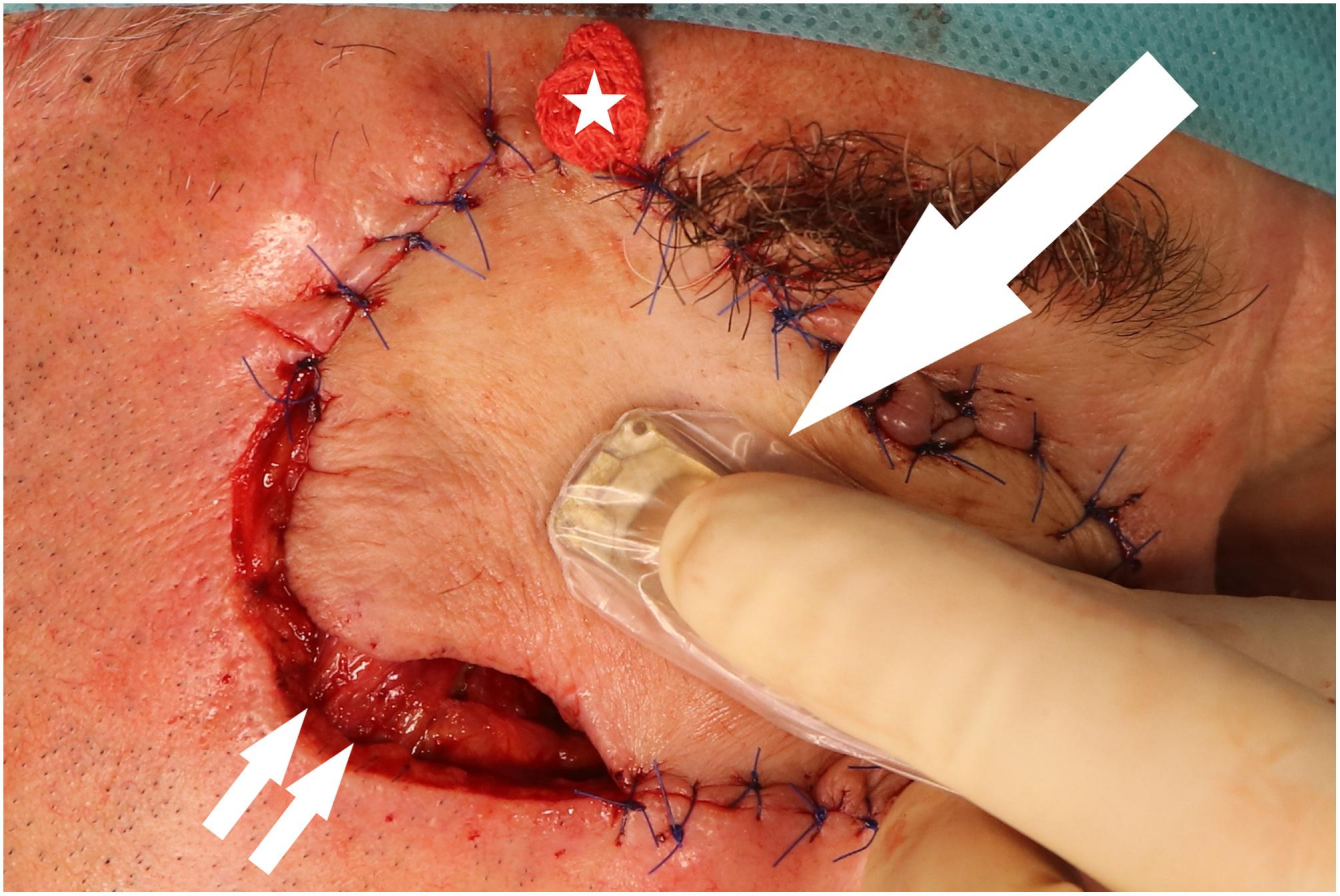
Flap perfusion measurement. Measurement of perfusion of a radial free forearm flap with the surface probe placed centrally on the dried skin portion of the flap in a sterile cover in a patient reconstructed in the right orbital region. Markings: white arrow = surface probe, double white arrow = flap pedicle, white asterisk = wound packing.

Flap perfusion measurement data were obtained intraoperatively with the patients being under general anesthesia after anastomosis release (after flap inset and before wound closure) with the O2C analysis system (O2C Oxygen-to-see, LEA Medizintechnik, Giesen, Germany), with the surface probe placed centrally on the dried skin portion of the flap in a sterile cover (Figure 1). Using laser Doppler spectroscopy (830 nm; 30 mW), blood flow (AU) were determined by evaluating the Doppler shift of the laser light due to the erythrocyte movement as the product of erythrocyte quantity and velocity (7, 20). Using white light spectroscopy (500–800 nm; 50 W), hemoglobin concentration (AU) and hemoglobin oxygen saturation (%) were determined by rating the sum of light absorbances or evaluating the light color change in comparison to reference hemoglobin spectra, respectively (7, 20). The measurement was carried out once for 10 s (with a lead time of 4 s) at 8 and 2 mm tissue depth under ambient light compensation control.

### 2.6 Statistical analysis

Patients were categorized into patients without postoperative flap revision (as control group) and patients with postoperative flap revision (as revision group). Differences between study groups in clinical parameters were analyzed using chi-squared test, Fisher exact test or Freeman Halton test for categorical data and Mann Whitney test for metric data. Differences between study groups in perfusion measurement values, i.e., flap blood flow, hemoglobin concentration, and hemoglobin oxygen saturation, were analyzed using Mann Whitney test and significant differences between study groups were analyzed using multivariable regression analysis adjusted for mean arterial blood pressure and catecholamine dose administered. Receiver operating characteristics analysis were performed for the flap perfusion parameters, which showed significant and in multivariable analysis persisting differences between the study groups. Diagnostic accuracy and theoretical optimal cut-off values for predicting postoperative flap revision were determined by the area under the curve (AUC) and the Youden index, with the corresponding test parameters such as sensitivity, specificity, positive predictive value (PPV), and negative predictive value (NPV) calculated (21, 22). Values of *p* < 0.05 were considered statistically significant. The statistical analysis was performed using SPSS version 28 (SPSS, IBM, New York, USA).

## 3 Results

### 3.1 Study population characteristics

The study population consisted of 296 patients, i.e., 269 patients in the control group without postoperative flap revision and 27 patients in the revision group with postoperative flap revision (Table 1). The revision group differed only in flap survival compared to the control group (no flap loss), with 5 flap losses in the revision group (1 flap due to arterial insufficiency and 4 flaps due to venous congestion) (*p* < 0.001). In the revision group, 26 flap revisions were performed due to venous congestion (25 cases involving venous thrombosis and 1 case involving a vessel kinking) and 1 flap revision was performed due to arterial insufficiency (1 case involving arterial thrombosis).

**Table 1:** Clinical characteristics of the study groups.

Variable	Control (*n* = 269)	Revision (*n* = 27)	*p*-value
Sex (*n*)			
Male	134 (49.8%)	18 (66.7%)	0.095
Female	135 (50.2%)	9 (33.3%)	
Age (*years*)	65.0 (17.0)	61.0 (15.0)	0.218
BMI (*kg/m²*)	25.4 (6.6)	25.0 (4.4)	0.530
ASA (*n*)			
1 + 2	152 (56.5%)	16 (59.3%)	0.783
3 + 4	117 (43.5%)	11 (40.7%)	
Flap location (*n*)			
Tongue	61 (22.7%)	8 (29.6%)	0.486
Floor of mouth	53 (19.7%)	6 (22.2%)	
Mandible	32 (11.9%)	4 (14.8%)	
Maxilla + hard palate	39 (14.5%)	2 (7.4%)	
Cheek	26 (9.7%)	3 (11.1%)	
Soft palate	17 (6.3%)	3 (11.1%)	
Extraoral	41 (15.2%)	1 (3.7%)	
Arterial anastomosis recipient vessel (*n*)			
External carotid artery	8 (3.0%)	2 (7.4%)	0.577
Facial artery	138 (51.3%)	12 (44.4%)	
Lingual artery	5 (1.9%)	0 (0.0%)	
Superior thyroid artery	115 (42.8%)	13 (48.1%)	
Other artery*	3 (1.1%)	0 (0.0%)	
Venous anastomosis recipient vessel (*n*)			
Internal jugular vein	203 (75.5%)	23 (85.2%)	0.186
Internal jugular vein + other vein**	36 (13.4%)	4 (14.8%)	
Other vein***	30 (11.2%)	0 (0.0%)	
Surgery duration (min)	513.0 (165.0)	477.0 (160.0)	0.076
Flap ischemia duration (min)	100.0 (34.0)	97.0 (33.0)	0.757
Diabetes (*n*)			
No	232 (86.2%)	25 (92.6%)	0.551
Yes	37 (13.8%)	2 (7.4%)	
Arterial hypertension (*n*)			
No	151 (56.1%)	20 (74.1%)	0.072
Yes	118 (43.9%)	7 (25.9%)	
Smoking status (*n*)			
No	176 (65.4%)	16 (59.3%)	0.522
Yes	93 (34.6%)	11 (40.7%)	
Peripheral artery disease (n)			
No	263 (97.8%)	27 (100.0%)	1.000
Yes	6 (2.2%)	0 (0.0%)	
Prior neck dissection (*n*)			
No	236 (87.7%)	23 (85.2%)	0.759
Yes	33 (12.3%)	4 (14.8%)	
Prior neck irradiation (*n*)			
No	257 (95.5%)	25 (92.6%)	0.371
Yes	12 (4.5%)	2 (7.4%)	
Flap survival (*n*)			
No	0 (0.0%)	5 (18.5%)	**<0**.**001**
Yes	269 (100.0%)	22 (81.5%)	

Parameters are indicated as numbers (with percentage) for categorical data or median (with interquartile range) for metric data separately for the control group (without postoperative flap revision) and the revision group (with postoperative flap revision); *p*-values corresponding to testing for differences between groups with chi-squared test (sex, ASA, arterial hypertension, smoking status), Fisher exact test (diabetes, peripheral artery disease, prior neck dissection, prior neck irradiation, flap survival), Freeman Halton test (flap location, arterial anastomosis recipient vessel, venous anastomosis recipient vessel), and Mann Whitney test (age, BMI, surgery duration, flap ischemia duration); significant *p*-values are bold; *occipital artery (*n* = 1), posterior auricular artery (*n* = 1), superficial temporal artery (*n* = 1); **facial vein (*n* = 10), external jugular vein (*n* = 9), retromandibular vein (*n* = 8), anterior jugular vein (*n* = 5), superior thyroid vein (*n* = 4), hypoglossal vein (*n* = 4); ***facial vein (*n* = 21), retromandibular vein (*n* = 4), superior thyroid vein (*n* = 3), external jugular vein (*n* = 1), superficial temporal vein (*n* = 1); abbreviations: BMI, body mass index; ASA, American society of Anesthesiologists score.

### 3.2 Intraoperative flap perfusion measurements values

**Figure 2:**
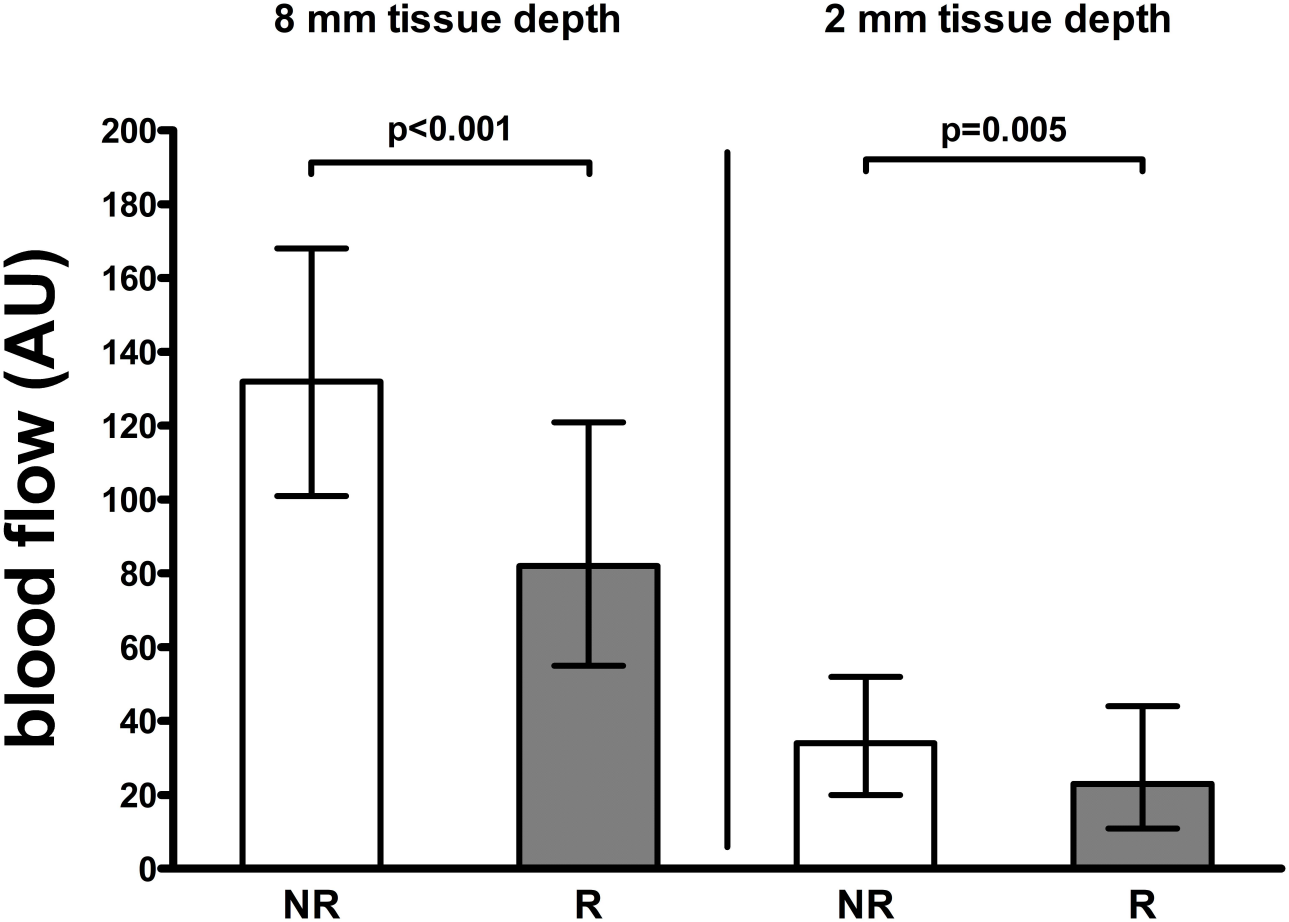
Blood flow measurement. Data shown as median (with interquartile range) for blood flow measurement (AU) for the control group (without postoperative flap revision) (*n* = 269) [NR] and the revision group (with postoperative flap revision) (*n* = 27) [R] [separately shown for 8 mm tissue depth (left) and 2 mm tissue depth (right)]; *p*-values corresponding to testing for differences between groups with Mann Whitney test; abbreviations: AU, arbitrary units; NR, no revision; R, revision.

**Figure 3:**
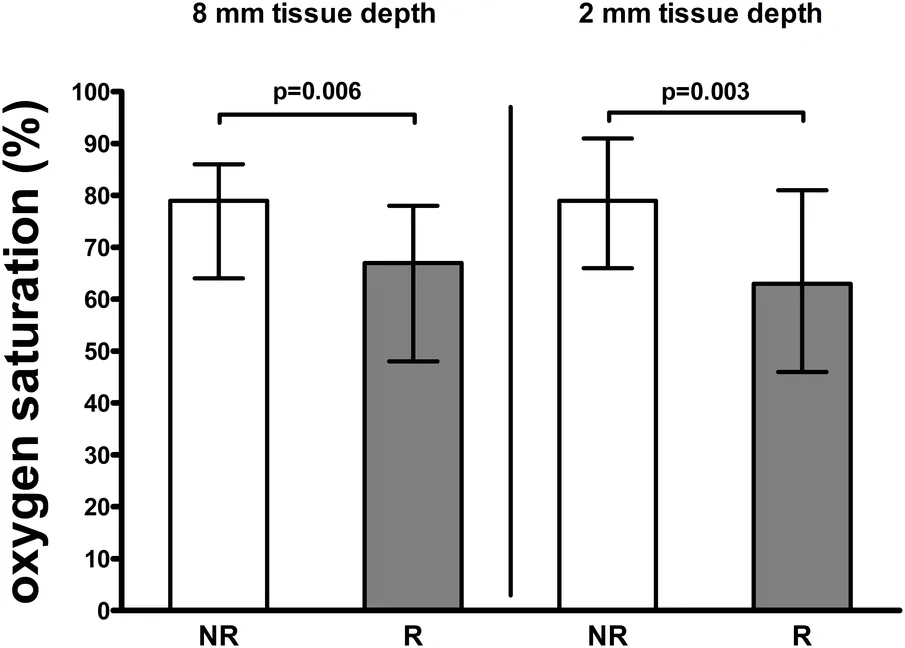
Hemoglobin oxygen saturation measurement. Data shown as median (with interquartile range) for hemoglobin oxygen saturation measurement (%) for the control group (without postoperative flap revision) (*n* = 269) [NR] and the revision group (with postoperative flap revision) (*n* = 27) [R] [separately shown for 8 mm tissue depth (left) and 2 mm tissue depth (right)]; *p*-values corresponding to testing for differences between groups with Mann Whitney test; abbreviations: AU, arbitrary units; NR, no revision; R, revision.

**Figure 4:**
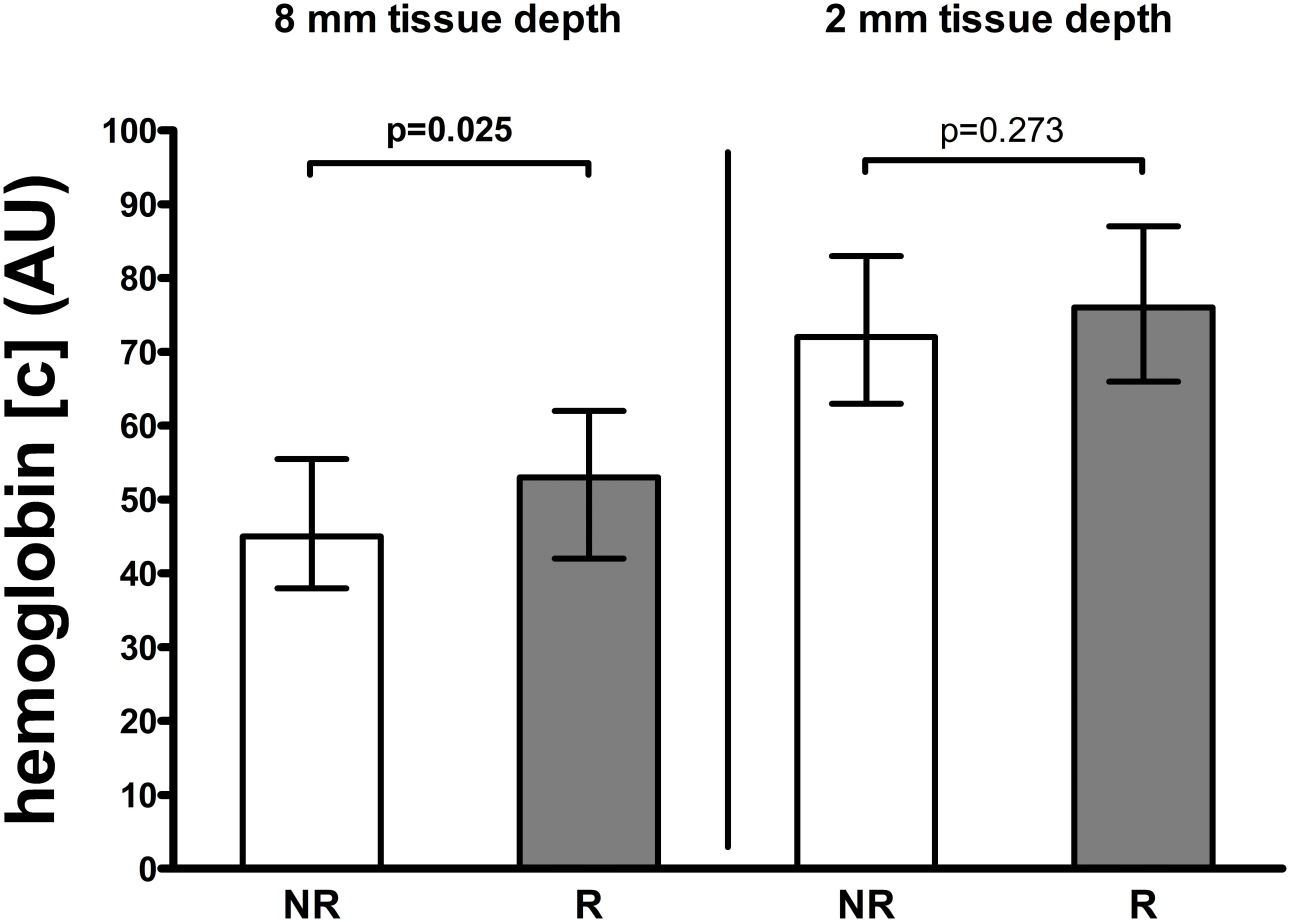
Hemoglobin concentration measurement. Data shown as median (with interquartile range) for hemoglobin concentration measurement (AU) for the control group (without postoperative flap revision) (*n* = 269) [NR] and the revision group (with postoperative flap revision) (*n* = 27) [R] [separately shown for 8 mm tissue depth (left) and 2 mm tissue depth (right)]; *p*-values corresponding to testing for differences between groups with Mann Whitney test; abbreviations: AU, arbitrary units; NR, no revision; R, revision.

The revision group had lower blood flow and hemoglobin oxygen saturation at 8 and 2 mm tissue depth than the control group (82.0 AU vs. 132.0 AU, *p* < 0.001; 23.0 AU vs. 34.0 AU, *p* = 0.005; 67.0% vs. 79.0%, *p* = 0.006; and 63.0% vs. 79.0%, *p* = 0.003) (Table 2, Figure 2, Figure 3). The revision group had a higher hemoglobin concentration at 8 mm tissue depth than the control group (53.0 AU vs. 45.0 AU, *p* = 0.025) (Table 2, Figure 4). These differences persisted in multivariable analysis (*p* < 0.001, *p* = 0.013, *p* = 0.006, *p* < 0.001, and *p* = 0.035).

**Table 2:** Comparison of flap perfusion measurements between the study groups.

Variable	Control (*n* = 269)	Revision (*n* = 27)	*p*-value
Blood flow 8 mm (AU)	132.0 (67.0)	82.0 (66.0)	**<0**.001*
Blood flow 2 mm (AU)	34.0 (32.0)	23.0 (33.0)	**0**.005*
Hemoglobin [c] 8 mm (AU)	45.0 (17.5)	53.0 (20.0)	**0**.025*
Hemoglobin [c] 2 mm (AU)	72.0 (20.0)	76.0 (21.0)	0.273
Oxygen saturation 8 mm (%)	79.0 (22.0)	67.0 (30.0)	**0**.006*
Oxygen saturation 2 mm (%)	79.0 (25.0)	63.0 (35.0)	**0**.003*

Parameters are indicated as median (with interquartile range) for blood flow, hemoglobin concentration, and hemoglobin oxygen saturation in 8 and 2 mm tissue depth separately for the control group (without postoperative flap revision) and the revision group (with postoperative flap revision); *p*-values corresponding to testing for differences between groups with Mann Whitney test; significant *p*-values are bold; *p < 0.05 upon adjustment for mean arterial blood pressure (mmHg) and administered catecholamine dose (µg/kg/min) in multiple regression analysis [blood flow 8 mm: *p* < 0.001; blood flow 2 mm: *p* = 0.013; hemoglobin (c) 8 mm: *p* = 0.035; oxygen saturation 8 mm: *p* = 0.006; oxygen saturation 2 mm: *p* < 0.001]; abbreviations: AU,  arbitrary units.

### 3.3 Cut-off values for predicting postoperative flap revision

The cut-off values for the prediction of postoperative flap revision were <82.5 AU {AUC 0.770 [95% confidence interval (CI) 0.670–0.871], *p* < 0.001, sensitivity 56%, specificity 90%, PPV 35%, NPV 95%} and <27.5 AU [AUC 0.665 (CI 0.548–0.782), *p* = 0.006, sensitivity 70%, specificity 61%, PPV 15%, NPV 95%] for flap blood flow at 8 and 2 mm tissue depth, >51.5 AU [AUC 0.631 (CI 0.531–0.731), *p* = 0.010, sensitivity 56%, specificity 69%, PPV 15%, NPV 94%] for hemoglobin concentration at 8 mm tissue depth, and <78.5% [AUC 0.659 (CI 0.554–0.765), *p* = 0.003, sensitivity 78%, specificity 52%, PPV 14%, NPV 96%] and <65.5% [AUC 0.674 (CI 0.565–0.784), *p* = 0.002, sensitivity 59%, specificity 75%, PPV 20%, NPV 95%] for hemoglobin oxygen saturation at 8 and 2 mm tissue depth (Table 3).

**Table 3:** Cut-off values for predicting postoperative flap revision.

Variable	AUC	*p*-value	Cut-off	YI	Sensitivity	Specificity	PPV	NPV
Blood flow								
8 mm	0.770 (0.670–0.871)	<0.001	<82.5 AU	0.451	56%	90%	35%	95%
2 mm	0.665 (0.548–0.782)	0.006	<27.5 AU	0.317	70%	61%	15%	95%
Hemoglobin [c]								
8 mm	0.631 (0.531–0.731)	0.010	>51.5 AU	0.247	56%	69%	15%	94%
Oxygen saturation								
8 mm	0.659 (0.554–0.765)	0.003	<78.5%	0.298	78%	52%	14%	96%
2 mm	0.674 (0.565–0.784)	0.002	<65.5%	0.347	59%	75%	20%	95%

Receiver operator characteristics analyses for blood flow in 8 and 2 mm tissue depth, hemoglobin concentration in 8 mm tissue depth, and oxygen saturation in 8 and 2 mm tissue depth based on data for the control group (without postoperative flap revision) (*n* = 269) and the revision group (with postoperative flap revision) (*n* = 27); theoretical optimal cut-off values were determined by calculation of the Youden Index (YI); abbreviations: AU, arbitrary units, AUC, area under the curve, YI, youden index, PPV, positive predictive value, NPV, negative predictive value.

## 4 Discussion

This retrospective study assessed whether intraoperative flap perfusion in RFFFs is associated with postoperative flap revision and cut-off values for flap perfusion can be determined to predict postoperative flap revision, since postoperative flap monitoring for timely detection and correction of vascular flap compromise is time-consuming, still controversial in terms of its frequency, and inevitably leads to an additional surgical procedure for flap revision (5, 6, 10–12). In contrast, the prediction of postoperative flap revision in the intraoperative setting could facilitate that intraoperatively the anastomosis could be checked and, if necessary, revised in flaps at risk to avoid postoperative flap revision (1, 4, 13–16, 23, 24). In addition, postoperatively the extent of flap monitoring could be adjusted towards a higher frequency in flaps at risk to detect and correct vascular flap compromise earlier and thus support flap salvage (1, 4, 15, 16, 24).

This study revealed that intraoperative flap perfusion was associated with postoperative flap revision, with flaps that underwent postoperative flap revision showing lower intraoperative blood flow and hemoglobin oxygen saturation and higher hemoglobin concentration. Moreover, postoperative flap revision could partially be predicted based on intraoperative flap perfusion measurement with respect to cut-off values for blood flow, hemoglobin concentration, and hemoglobin oxygen saturation.

The observation that intraoperatively measured blood flow and hemoglobin oxygen saturation at 8 and 2 mm tissue depth were lower and hemoglobin concentration at 8 mm tissue depth was higher in flaps with postoperative flap revision is consistent to postoperative monitoring with the O2C analysis system described in previous studies (7, 8). That also applies for the observation that cut-off values for intraoperative flap perfusion were minimum thresholds for blood flow and hemoglobin oxygen saturation and a maximum threshold for hemoglobin concentration (7, 8). These previous studies have demonstrated that lower blood flow and hemoglobin oxygen saturation and higher hemoglobin concentration are associated with flap failure and that threshold values for blood flow and hemoglobin oxygen saturation were absolute minimum values and for hemoglobin concentration relative maximum values (7, 8). However, the lower blood flow and hemoglobin oxygen saturation in flaps with postoperative flap revision observed in this study is interesting, as almost all flap revisions in this study were conducted due to venous congestion, a condition in which, at least initially, only a higher hemoglobin concentration would have been expected (7, 8). The lower blood flow and hemoglobin oxygen saturation also in flaps with venous congestion could potentially be due to a veno-arteriolar response, which reduces arterial inflow into the flap tissue when venous pressure is elevated due to venous congestion (7, 8, 25). However, it remains questionable why this observation can be made at this early stage after flap reperfusion in the intraoperative setting. Besides, it should be noted that intraoperative differences in flap perfusion between flaps with and without postoperative flap revision and related cut-off values for the prediction of postoperative flap revision are in contrast to a previous study, which found only postoperative decreasing trends to be associated with postoperative flap revision (17). However, these contrary results might be attributable to differences in study cohorts (e.g., patient demographics) or study methods (e.g., measurement timing). In general, an explanation for the altered perfusion parameters in flaps with postoperative flap revision may be that these flaps already intraoperatively had clinically and, with regard to the flap perfusion thresholds that had been defined in previous studies as indicators of vascular flap compromise, technically inapparent vascular flap compromise (7, 8). This inapparent vascular flap compromise could lead to an increased hemoglobin concentration as well as a decreased flap blood flow and consequently hemoglobin oxygen saturation, as both perfusion parameters are positively associated (7, 8).

Regarding the determination of cut-off values for predicting postoperative flap revision based on receiver operating characteristics analysis, it should be noted that the related test parameters, e.g., sensitivity and specificity, were partially low (21, 22). These test parameters could be interpreted in the light of the minimum additional time required intraoperatively to check and, if necessary, revise the anastomosis and the modest additional time required postoperatively to monitor the flap more frequently on the one hand and the devastating consequences of flap loss on the other hand (5, 6, 10, 13, 14). However, the limited predictive performance of the cut-off values hampers their usefulness in clinical practice. This becomes particularly evident in the low positive predictive values, which results in many flaps being falsely classified as flaps at risk, thereby unnecessarily prolonging the duration of surgery and creating unnecessary additional workload.

Limitations of the study include the lack of consideration of all variables potentially influencing flap perfusion and the need for flap revision, e.g., the flap pedicle length and diameter (11, 26–28). Moreover, variability in intraoperative flap perfusion, e.g., due to general perfusion variation within the flap or differences among the flaps in terms of the time interval between reperfusion of the flap and the measurement of flap perfusion, could potentially lead to a bias in the study results. In this context, measuring intraoral flaps may have been more challenging due to limited access to the oral cavity, and the potential influence of systemic parameters, e.g., blood hemoglobin concentration and oxygenation, on the measured perfusion values cannot be ruled out. In addition, no preoperative reference values for flap perfusion prior to flap harvesting were available, which cannot be changed due to the retrospective nature of the study. In general, the low flap revision rate in this study, which can also be observed in the literature, lessens the strength of this study with regard to the derivation of cut-off values (2, 4). It should be noted that flaps that were revised due to arterial insufficiency were underrepresented in this study, and that the cut-off values were not validated, which underscores the exploratory nature of the study results and limits their generalizability. In this context, the single-center study design, including center-specific surgical procedures and perioperative management protocols, further limits the broader applicability of the study results. In general, there is a risk of incorporation bias in this study, as flap perfusion was used both as the parameter under investigation and as the parameter used for the decision to perform flap revision as the outcome measure.

This exploratory study demonstrated that flaps that had to be revised postoperatively already had lower blood flow and hemoglobin oxygen saturation and higher hemoglobin concentration values in intraoperative flap perfusion measurement (though these values did not indicate vascular flap compromise based on established thresholds used with the O2C analysis system). It also demonstrated that postoperative flap revision could partially be predicted based on flap perfusion cut-off values. However, the reduced generalizability of the study findings due to the single-center study design, the limited validity of the endpoint (flap revision) due to potential incorporation bias, and the unnecessary additional workload resulting from low positive predictive values should be acknowledged when interpreting the study findings and assessing their clinical implications. These findings need to be confirmed in prospective studies that account for potentially confounding variables, such as systemic blood parameters, to represent an initial step towards the prediction of postoperative flap revision. This may both support the decision to check and revise the anastomosis in flaps at risk intraoperatively and guide the adjustment of flap monitoring in flaps at risk postoperatively (1, 4–6, 13–16, 23, 24).

## 5 Conclusion

This exploratory study demonstrated that intraoperative flap perfusion and postoperative flap revision were associated, with flaps undergoing postoperative flap revision already showing a lower blood flow and lower hemoglobin oxygen saturation as well as a higher hemoglobin concentration in the intraoperative flap perfusion measurement. Based on cut-off values for flap perfusion, i.e., blood flow <82.5 AU, hemoglobin concentration >51.5 AU, and hemoglobin oxygen saturation <78.5%, postoperative flap revision could partially be predicted. Prospective studies with larger study cohorts are required to confirm these findings, which could support the implementation of prevention strategies such as intraoperative anastomosis check and closer postoperative flap monitoring for flaps at risk.

## Data Availability

The datasets presented in this article are not readily available because the datasets generated and analyzed during the current study are under further analysis and are available from the corresponding author on reasonable request. Requests to access the datasets should be directed to mark.ooms@rwth-aachen.de.
